# Reply to "Comment on 'K-Alpha calculator, Krippendorff's Alpha calculator: A user-friendly tool for computing Krippendorff's Alpha inter-rater reliability coefficient': Reconciling the ratio-scale computational implementation"

**DOI:** 10.1016/j.mex.2026.104168

**Published:** 2026-09-19

**Authors:** Giacomo Marzi, Marco Balzano, Davide Marchiori

**Affiliations:** a IMT School for Advanced Studies Lucca, Lucca, Italy; b Department of Economics, Management, Mathematics and Statistics, University of Trieste, Trieste, Italy; c Center for Behavioral Institutional Design, New York University Abu Dhabi, UAE

We thank MethodsX for bringing to our attention the issue identified by Abdullah and colleagues, and for the opportunity to respond (see the letter "Comment on 'K-Alpha calculator, Krippendorff's Alpha calculator: A user-friendly tool for computing Krippendorff's Alpha inter-rater reliability coefficient': Reconciling the ratio-scale computational implementation" published in MethodsX). The issue is confined to a specific part of the ratio-scale computation, while the nominal, ordinal, and interval computations are unaffected and require no change. Ratio-scale computations on datasets whose minimum value is 1 are likewise unaffected.

The ratio-scale implementation has now been fully updated in release 2.1, currently available at https://k-alpha.org.The release 2.1 is archived on Figshare (https://doi.org/10.6084/m9.figshare.24847560.v5) and maintained on GitHub (https://github.com/davide-marchiori/k-alpha).

We thank the growing community of K-Alpha Calculator users for their continued use and feedback, and we welcome further comments through the public GitHub repository, where a record of updates is maintained. The K-Alpha Calculator will remain freely available and open source, and we are committed to its ongoing maintenance and improvement..

## Data Availability

Data will be made available on request.

